# Neuroleptic malignant syndrome following catatonia: Vigilance is the price of antipsychotic prescription

**DOI:** 10.1177/2050313X17695999

**Published:** 2017-03-31

**Authors:** Thomas J Reilly, Sean Cross, David M Taylor, Richard Haslam, Sophie C Tomlin, Benjamin Gaastra

**Affiliations:** 1 Department of Psychosis Studies, Institute of Psychiatry, Psychology & Neuroscience, King’s College London, London, UK; 2 Ladywell Unit, South London and Maudsley NHS Foundation Trust, London, UK; 3 Department of Psychological Medicine, King’s College Hospital, South London and Maudsley NHS Foundation Trust, London, UK; 4 Pharmaceutical Sciences Clinical Academic Group, King’s Health Partners, South London and Maudsley NHS Foundation Trust, London, UK

**Keywords:** Catatonia, schizophrenia, antipsychotic agents, neuroleptic, malignant syndrome

## Abstract

**Objectives:**

To describe a case of neuroleptic malignant syndrome following antipsychotic treatment of catatonia, highlighting the potentially serious complications of this rare adverse drug reaction.

**Methods:**

We present a case report of a patient who developed this syndrome with various sequelae.

**Results:**

The patient developed neuroleptic after being treated with lorazepam and olanzapine for catatonia. He subsequently developed the complications of rhabdomyolysis, acute kidney injury, pulmonary embolism, urinary retention and ileus. He received high-dose lorazepam, anticoagulation and intravenous fluids. Antipsychotic medication in the form of haloperidol was reinstated with no adverse effect, and he went on to make a full recovery.

**Conclusions:**

This case illustrates the potential life-threatening complications of neuroleptic malignant syndrome and the need for a low index of clinical suspicion. It also highlights the lack of evidence for treatment of catatonia, including the use of antipsychotics.

## Introduction

Neuroleptic malignant syndrome (NMS) is a rare but life-threatening reaction to antipsychotics. It commonly presents with pyrexia, autonomic instability, rigidity, and altered consciousness^1^ and can be precipitated by any type of antipsychotic drug.^2^ NMS shares similar clinical features and treatments with catatonia, and it has been argued that these are two conditions on the same spectrum. In particular, a form of catatonia with pyrexia and autonomic instability (*lethal* or *malignant* catatonia) is thought to be indistinguishable from NMS.^3^ In contrast, others have divided the two entities based on their pathophysiology, with NMS described as a subcortical ‘motor syndrome’ caused by dopaminergic dysregulation and catatonia being due to GABAergic dysregulation resulting in a cortical ‘psychomotor syndrome’.^4^ Cases of catatonic symptoms preceding NMS have been widely reported, leading some to suggest catatonia is a risk factor for NMS.^5^ Here, we present a case of NMS and subsequent complications in a patient who initially presented with catatonia.

## Case report

A 49-year-old British man, of African ethnicity, presented to the emergency department with rigidity, negativism, immobility and mutism which had worsened over the previous 2 days. There were no prominent affective features and no history of illicit drug use. He had a history of schizophrenia but no prior catatonic symptoms. He had previously been treated with antipsychotics, most recently haloperidol, but on presentation had been medication-free for 7 months. The initial diagnosis was a relapse of schizophrenia with catatonic symptoms. He showed a partial response to 1 mg of lorazepam, so was treated with this three times daily. There was no substantial improvement; therefore, on Day 3, the decision was made to commence antipsychotic medication. Over the next 48 hours, he received a total of 20 mg of olanzapine. On Day 5, he became pyrexial, tachycardic and had diaphoresis (see Table 1 for clinical parameters). He was quickly transferred to a medical ward for further management.

**Table 1.** Clinical observations.

Parameter	Day 1	Day 5	Day 6	Day 7	Day 8	Day 12	Day 18	Day 25	Day 53 (discharge)
Tympanic temperature (°C)	36.8	38.9	38.8	39.3	38.1	38.6	36.7	36.7	36.6
Respiratory rate	17	22	18	18	17	18	20	18	18
Peripheral oxygen saturations (%)	97	94	100	97	94	99	90	98	98
Pulse (bpm)	80	104	103	100	104	110	89	64	78
Blood pressure (mmHg)	158/86	186/106	155/90	156/99	174/111	149/97	150/96	140/95	102/64

On arrival in the medical ward, he was non-communicative with increased tone throughout. Blood tests revealed acute kidney injury (AKI), deranged liver function tests (LFTs) and raised creatine kinase (CK; see Table 2). A septic screen (chest X-ray, blood and urine cultures) was negative. Intravenous fluids were commenced and regular lorazepam continued. He had multiple reviews by the Intensive Care Unit team but remained on a medical ward; 7 days after first presenting, he developed urinary retention, requiring catheterisation. Blood tests showed a CK of 21,000 µ/L, deranged LFTs and improved renal function. Lorazepam was increased to 6 mg per day. Over the next 4 days, blood tests showed a falling CK and improving LFTs. Lorazepam was gradually increased to 12 mg daily by Day 13. On Day 16, he was apyrexial and showed clinical improvement, becoming less rigid and speaking some words. However, he developed a distended abdomen and it was noted that he had only defaecated once since admission. Abdominal computed tomography (CT) showed no mechanical obstruction; a diagnosis of ileus was made. On Day 19, peripheral oxygen saturations fell to 90%. A chest X-ray showed no evidence of infection and a CT pulmonary angiogram on Day 22 showed a right lower lobe pulmonary embolism. Treatment dose low-molecular-weight heparin was commenced (he had been on prophylactic dose prior to this). Over Days 23 and 24, he began to speak in sentences. The dose of lorazepam was increased to 20 mg per day; he tolerated the high dose of benzodiazepine without adverse effect. Over the next 11 days, he improved clinically with resolution of abdominal distension and a reduction in rigidity. For the first time since being admitted to the medical ward, he was able to mobilise and engage in conversation.

**Table 2.** Venous blood parameters.

Parameter	Normal range	Day 1	Day 6	Day 10	Day 13	Day 16	Day 19	Day 30	Day 53 (discharge)
WCC	4.0–11.0 × 10^9^ L^−1^	5.8	7.86	7.58	8.91	9.12	6.29	7	5.4
CK	40–235 µ/L	1139	21,769	2800	27,208	4464	2650	216	97
Potassium	3.6–5.0 mmol/L	3.8	4.3	4.1	3.5	4.3	3.6	3.6	4.2
Sodium	136–145 mmol/L	145	152	145	143	138	140	137	138
Creatinine	75–122 µmol/L	102	141	74	94	74	66	60	75
Urea	2.0–7.8 mmol/L	5	13.1	7.3	7.1	3.2	4.4	3.8	3.6
eGFR	>90 mL/min/1.73 m^2^	67	46	>90	74	>90	>90	>90	>90
CRP	2–7 mg/L	<5	22.6	10.6	29	54.4	38.9	32.7	<5
AST	10–40 IU/L		420	112		110	65	25	
Alk phos	36–126 IU/L		74	50	71	65	70	82	65
Bilirubin	2–22 µmol/L		21	22	15	12	10	32.7	5

WCC: white cell count; CK: creatine kinase; eGFR: estimated glomerular filtration rate; CRP: C-reactive protein; AST: aspartate transaminase; alk phos: alkaline phosphatase.

He was discharged back to a psychiatric ward on Day 36 on a reducing regime of lorazepam. Haloperidol was recommenced at a low dose of 2.5 mg, as he had previously used this medication for maintenance treatment with no adverse effect. Anticoagulation with warfarin replaced low-molecular-weight heparin. His blood parameters returned to normal, he returned to baseline function and was discharged to community services. Haloperidol was later increased to 5 mg per day. In the year following discharge, there was no relapse in terms of catatonia or other psychotic symptoms.

## Discussion

This case adds to a large body of previous case reports of NMS following antipsychotic treatment for schizophrenia with catatonic symptoms.^6^ It also shows the varying complications that can accompany NMS. These complications have been reported previously, but not, to our knowledge, simultaneously in one patient. As many of these complications are life-threatening, clinicians should have a low index of suspicion for NMS.

In a recent large (n = 1346) record-linkage study in the United States, the case fatality rate for NMS was 5.6%.^7^ The most common complication was rhabdomyolysis (30.1%), while age, acute respiratory failure, AKI, sepsis, and comorbid congestive heart failure were predictors of mortality. Pulmonary embolism was a complication in only 1% of patients with NMS. Of note, the patient we present developed pulmonary embolism despite being given prophylactic low-molecular-weight heparin. AKI occurred in 17.7% patients with NMS, and of these patients, 5.9% required haemodialysis. For the case presented here, rhabdomyolysis and dehydration were likely contributors to AKI, which was treated effectively with intravenous fluids and did not require dialysis. The ileus and urinary retention observed in this case are complications which have not previously featured in case reports. However, they can be explained in the context of a severe illness with dehydration and multi-organ dysfunction. They occurred several days after the withdrawal of olanzapine and therefore are unlikely to be attributable to the anticholinergic effects of this medication.

The majority of clinical research linking the development of NMS with catatonia is anecdotal. Similarly, there is a dearth of evidence for the treatment of catatonic symptoms in the context of schizophrenia. A Cochrane Review found no suitable randomised controlled trials (RCT) for the use of benzodiazepines in this situation.^8^ Since then, there has been a negative randomised placebo-controlled cross-over trial of amineptine for chronic catatonic symptoms in schizophrenia.^9^ The current Maudsley Prescribing Guidelines recommend the use of second-generation antipsychotics as first-line for catatonia in schizophrenia (when NMS has been ruled out), with high dose of benzodiazepines and electroconvulsive therapy (ECT) being further treatment options.^10^ Whether one or a combination of these therapeutic options is most effective has yet to be established empirically. With this current uncertainty, clinicians could be justified in gauging response to benzodiazepines in people with schizophrenia and catatonic symptoms, irrespective of antipsychotic use. Notably, there is one RCT registered for the use of ECT compared with medication for catatonia currently registered (https://clinicaltrials.gov/). This case serves as a reminder that high dose of benzodiazepines can be tolerated safely. Indeed, the Maudsley Prescribing Guidelines^10^ recommend up to 24 mg per day for the treatment of catatonia, which is considerably higher than the usual clinical dose for other conditions.

Antipsychotic rechallenge with haloperidol 2.5 mg was successful in this case. However, others have suggested that the choice of antipsychotic should be one with low D2 affinity.^11^ It is important to wait for resolution of NMS before rechallenge with some suggesting a ‘washout’ period of 2 weeks.^11^ It is also advisable to restart at a low dose and to monitor for re-emerging signs of NMS.

## Conclusion

In summary, this case reports highlights the many severe complications that occurred when a gentleman presenting with catatonia was treated with olanzapine. Clinicians should monitor for the autonomic instability associated with NMS when prescribing antipsychotics, particularly when patients have catatonic symptoms. There is a lack of empirical clinical evidence for the use of antipsychotics in catatonia, with decisions currently based on expert opinion.
